# RCNet: A Hybrid Network Adapted for OCTA Retinal Capillary Segmentation

**DOI:** 10.3390/bioengineering13101147

**Published:** 2026-09-30

**Authors:** Xudong Wang, Qinghong Gao, Ran Yang, Xuelian Yang, Rui Yao, Shu’an Liu, Maosong Jiang, Caiye Fan, Zuoping Tan, Yuanyuan Wang

**Affiliations:** 1 School of Ophthalmology & Optometry, Wenzhou Medical University, Wenzhou 325000, China; 2 School of Data Science and Artificial Intelligence, Wenzhou University of Technology, Wenzhou 325000, China

**Keywords:** deep learning, OCTA, microvasculature segmentation

## Abstract

Optical Coherence Tomography Angiography (OCTA) provides clear visualization of ocular microvascular details, serving as a critical tool for assessing retinal and choroidal vascular systems. However, the complex capillary structure and low capillary-to-background contrast in high-resolution OCTA images pose significant challenges for segmentation. To address the low segmentation accuracy of traditional methods, this paper proposes RCNet, a hybrid CNN-Transformer network. RCNet adopts an encoder–decoder architecture based on Bi-Level Routing Attention (BRA), where bi-level routing attention focuses on densely vascularized regions, optimizing global capillary feature extraction. A Convolutional Bottleneck Module (CBM) splits bottleneck features into two channel groups using a fixed ratio α. A high-capacity branch enhances local details, while a lightweight branch preserves complementary information. The branches are then fused. Additionally, a Dense Skip Connection Module (DSCM) enhances feature information flow in low-contrast regions, compensating for spatial information loss. By integrating CNN’s local feature extraction with Transformer’s global dependency modeling, RCNet achieves accurate segmentation of OCTA capillary images. Experiments on the OCTA-500 and ROSE datasets demonstrate that RCNet achieved a higher point estimate under the current protocol, facilitating and assisting in the clinical assessment of ocular diseases.

## 1. Introduction

**Figure 1:**
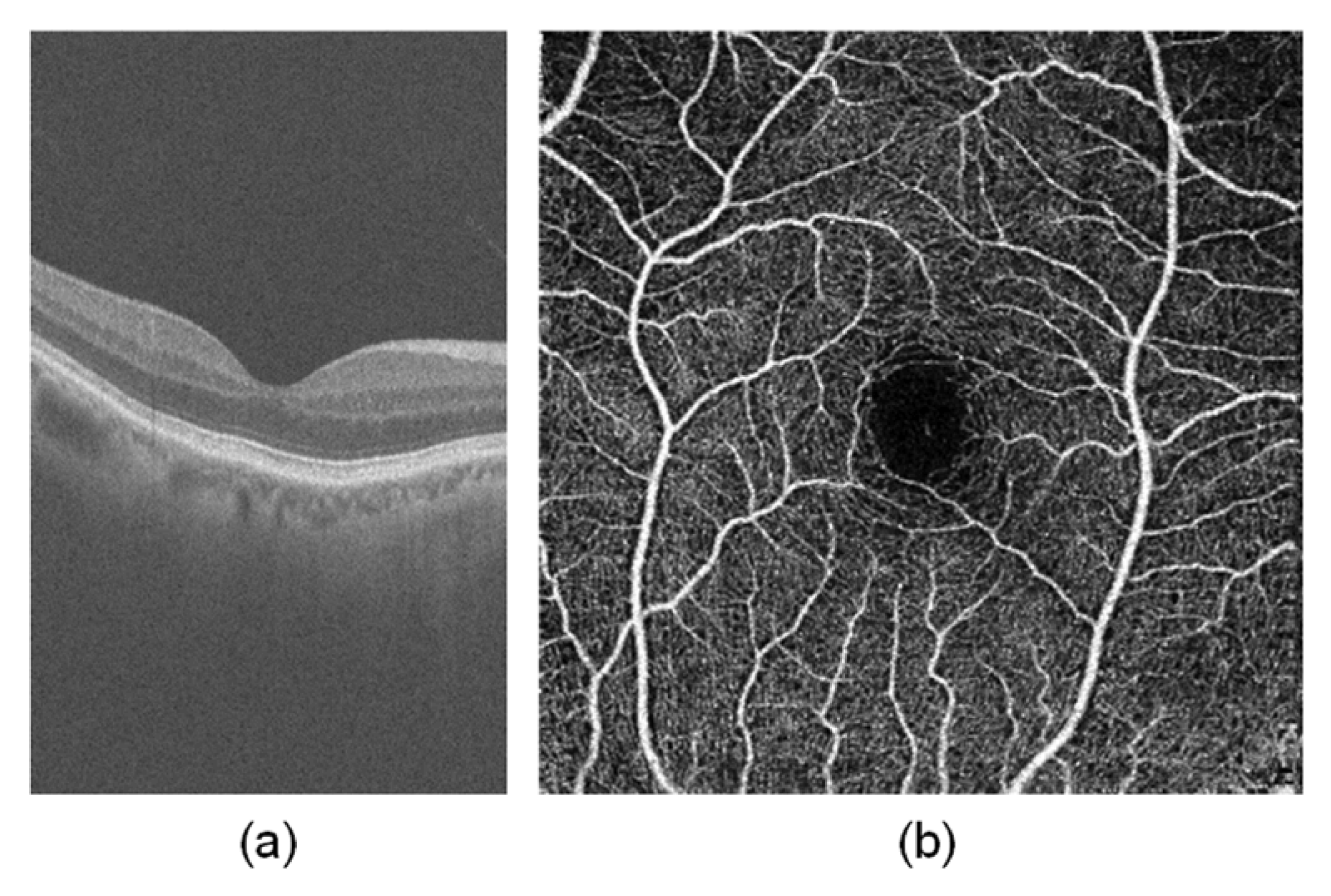
OCT image (**a**) and OCTA 2D en-face image (**b**) of the same eye.

Optical Coherence Tomography Angiography (OCTA) represents a significant extension and advancement of Optical Coherence Tomography (OCT) technology. OCTA provides novel non-invasive imaging modality in ophthalmic diagnostics, playing a pivotal role in both diagnosis and treatment [1]. As shown in Figure 1, while OCT provides high-resolution cross-sectional images of ocular tissues, OCTA captures dynamic information of retinal and choroidal microvasculature, visually demonstrating capillary structures and blood flow status [2]. While OCT imaging facilitates observation of ocular structures, OCTA delivers more precise and intuitive visualization of retinal capillaries, aiding in the diagnosis of capillary diseases such as age-related macular degeneration (AMD) and diabetic retinopathy (DR).

**Figure 2:**
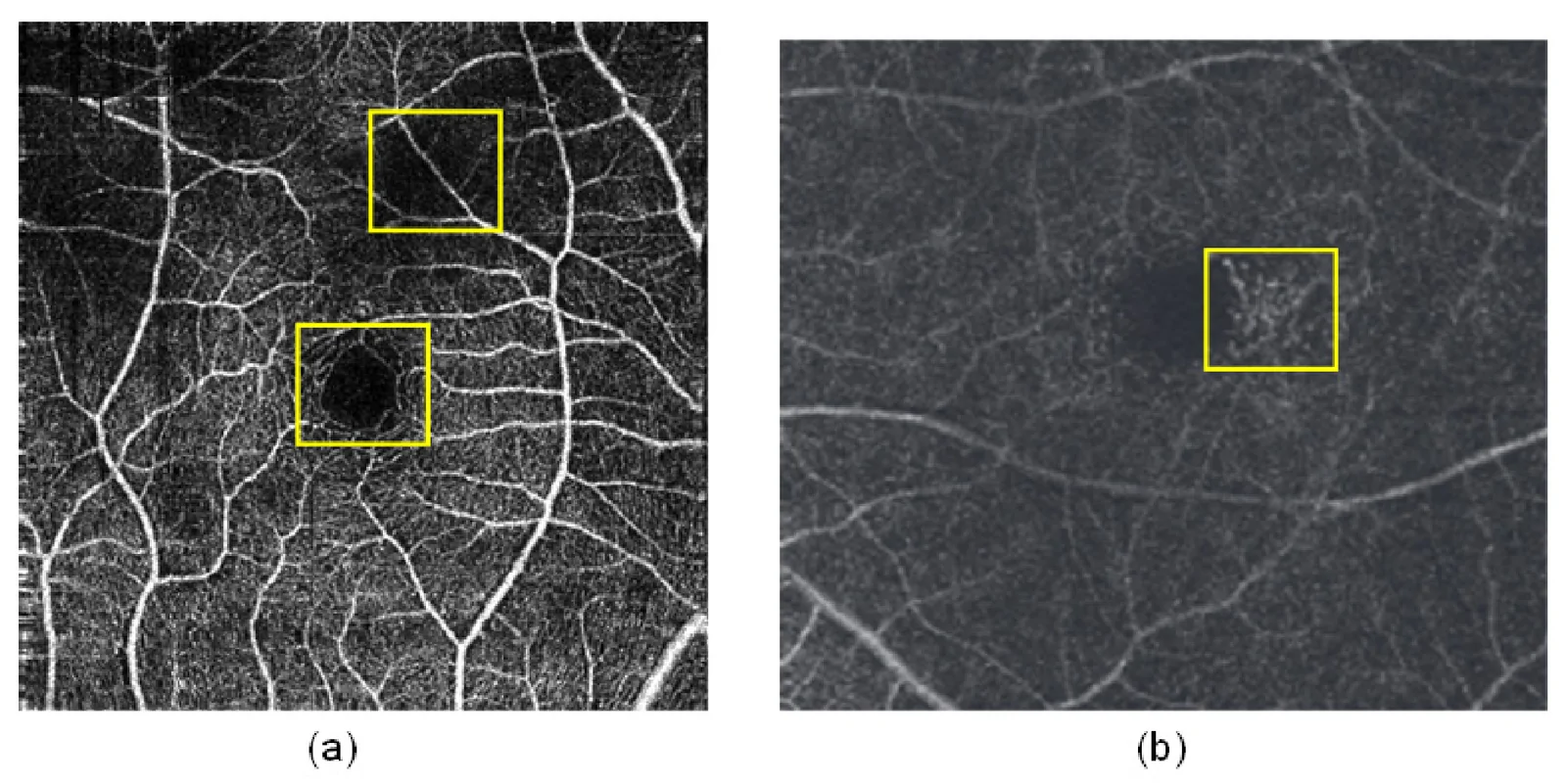
OCTA 2D en-face imaging results, with the image box highlighting localized tissue abnormalities, an age-related macular degeneration patient (**a**), a patient with type 2 macular capillary dilation (**b**).

Markers in OCTA imaging results hold significant clinical diagnostic value. As shown in Figure 1, OCTA imaging primarily consists of the foveal avascular zone (FAZ) and vascular zones. The fovea is a critical region for forming detailed visual function, while the FAZ ensures this function remains unaffected by surrounding vessels. The morphology and size of the FAZ, along with the density of surrounding capillaries, reflect the degree of macular ischemia. These findings are closely associated with various retinal capillary diseases [3]. Changes in FAZ area may indicate the onset of certain retinal pathologies—for instance, an enlarged FAZ suggests macular foveal ischemia, while neovascularization may lead to a reduced FAZ area. The retinal capillary region holds equal significance. Beyond common ophthalmic conditions like glaucoma, retinal neovascular diseases, and retinal vein occlusion—where OCTA can detect abnormal capillary morphology [4]—systemic diseases also correlate with retinal vasculature. For instance, reduced retinal capillary density in the macular region is observed in patients with Alzheimer’s Disease (AD) and Mild Cognitive Impairment (MCI) [5]. These structural alterations are detectable via OCTA. Figure 2 illustrates diagnostic outcomes using OCTA for ophthalmic conditions: Imaging results from an age-related macular degeneration patient (a) reveal an enlarged avascular zone in the fovea centralis and reduced capillary density in the superior region. Imaging results from a patient with type 2 macular capillary dilation (b) show microvascular abnormalities in the deep retinal capillary plexus [6]. These clinical observations underscore the importance of accurately delineating capillary structures in OCTA images, as precise segmentation is a prerequisite for reliable quantitative analysis and downstream clinical decision-making.

Despite its clinical significance, the automated segmentation of OCTA capillaries remains an open challenge because of two primary technical bottlenecks. First, the intricate and dense topology of microcapillaries requires models to maintain high structural continuity; however, standard architectures often produce fragmented results at complex bifurcations. Second, the extremely low vessel-to-background contrast and the presence of speckle noise in high-resolution scans frequently lead to the loss of weak vascular signals. Although current research has achieved success in FAZ segmentation, the pixel-accurate extraction of the entire capillary network is considerably more difficult, demanding a sophisticated balance between global context modeling and local detail refinement. Owing to the rapid advancement of artificial intelligence, computer vision has been widely applied in ophthalmic image analysis. Deep learning techniques have achieved substantial progress in the field of ophthalmic image segmentation [7]. To further improve the segmentation performance of OCTA 2D en-face retinal capillary images, this paper proposes a task-oriented hybrid CNN-Transformer architecture RCNet for image segmentation. The contributions of this study can be summarized as follows:

• We propose RCNet, a CNN-Transformer hybrid architecture for segmenting OCTA 2D en-face retinal capillary images, addressing the challenges of maintaining structural continuity in dense capillary networks and recovering weak vascular signals under low contrast and speckle noise, achieving improved segmentation accuracy.
• A DSCM module is introduced. Inserted between the decoder and encoder, this module integrates capillary morphological features of varying scales to achieve effective alignment between main vessel and branch microvascular features. This enhances the segmentation results’ naturalness and smoothness while improving capillary feature information flow in low-contrast regions, compensating for local spatial information loss during segmentation.
• A CBM is introduced at the bottleneck stage to enhance the local representation of capillary structures. Using a fixed channel-split ratio, CBM processes one channel group with higher-capacity convolutional operations and the complementary group with lightweight operations. The branch outputs are subsequently fused to combine their feature representations.

## 2. Related Work

### 2.1. Segmentation with Deep Learning

Traditional automatic segmentation systems based on edge detection and mathematical methods have limitations when handling complex scenarios. The emergence of deep learning approaches has significantly improved the quality and accuracy of image segmentation. U-Net [8], a convolutional neural network featuring a multi-scale encoder–decoder architecture, has been widely applied in medical image segmentation [9]. Inspired by U-Net, variants such as U-Net++ [10] by Zhou, Zongwei et al. and UNet3+ [11] by Huang, Huimin et al. have also demonstrated strong performance in medical image segmentation. Attention U-Net [12], proposed by Oktay, Ozan et al., employs attention mechanisms to suppress irrelevant regions in input images while highlighting salient features in specific local areas, further enhancing U-Net’s performance. The success of convolutional neural networks (CNNs) primarily stems from their specific inductive biases in handling scale invariance and modeling local visual structures within images. While this locality enhances CNN efficiency, it weakens their ability to capture long-range spatial dependencies [13], particularly for objects exhibiting significant variations in shape and texture [14]—such as retinal capillary images—thereby limiting CNN effectiveness in segmentation tasks [15].

The success of Transformers in natural language processing (NLP) [16] tasks spurred the development of Vision Transformers [17], which simultaneously overcame the limitations of CNNs. Vision Transformers (ViTs) utilize Multi-Head Self-Attention (MSA) to capture global context and establish long-range dependencies, achieving strong performance on ImageNet [18]. However, ViTs require substantial data for generalization and suffer from quadratic complexity issues. DeiT [19], proposed by Touvron, Hugo et al., aims to address these challenges.

Although the Transformer architecture can capture global dependencies, it exhibits weaker performance in accurately segmenting local image details, which affects precise boundary segmentation. Recent advancements in Vision Transformers emphasize the importance of multi-scale feature representations. CrossViT [20] and HRViT [21] introduce a dual-branch Transformer architecture to enhance contextual information extraction capabilities and improve semantic segmentation performance. In medical image segmentation, SwinUnet [22] applies Transformers to both encoder and decoder architectures to enable global feature representation across multiple levels. However, it exhibits poor performance in representing fine-grained features for complex organ segmentation tasks.

Transformers may overlook low-level features in images due to their self-attention mechanism, resulting in limitations when capturing local details in medical images. Hybrid CNN-Transformer architectures, such as TransUnet [23], address medical image segmentation by combining the locality of CNNs with the long-range dependencies of Transformers. However, they face challenges in efficiently integrating features from different levels, maintaining feature consistency, and correctly utilizing the multi-scale information obtained from hierarchical encoders.

### 2.2. Segmentation Tasks in OCTA

Unique markers detected in retinal capillary images through OCTA technology can play a role in the early diagnosis of various diseases. Accurate segmentation results can play a crucial role in disease damage visualization, disease monitoring and analysis, and assisting clinical diagnosis. A. Kashani et al. [24] proposed using a Markov random field model from machine learning to segment 2D en-face OCTA capillary images. However, the segmentation results exhibited significant overfitting, with poor segmentation performance reflecting the limitations of traditional methods in feature extraction. Zhijin et al. [25] proposed an end-to-end trained deep network for unsupervised FAZ segmentation of OCTA images. Lin, L. et al. [26] introduced the multi-level boundary shape and distance-aware joint learning framework BSDA-Net for FAZ segmentation and diagnostic classification from OCTA images. Ma et al. [27] introduced OCTA-Net, a two-stage coarse-to-fine framework. Although OCTA-Net effectively identifies large vessels, its Split-and-Reconstruct Network (SRN) primarily focuses on local patch-based refinement, which may overlook long-range topological connectivity, leading to ‘broken branches’ in terminal capillary areas. Li et al. [28] constructed a public OCTA image dataset named OCTA-500 and proposed a novel three-dimensional-to-two-dimensional image projection network (IPN) for segmenting FAZ and retinal vascular (RV) abnormalities. OCT2Former [29] combines dynamic token aggregation with an auxiliary convolutional branch to capture global vascular context while retaining local inductive bias. LA-Net [30] further exploits volumetric OCT/OCTA information through layer attention and a 3D-to-2D projection pathway, together with boundary-aware feature learning. Foundation-model adaptation has also been investigated in SAM-OCTA [31], which applies parameter-efficient fine-tuning and prompt-generation strategies to multiple OCTA segmentation tasks. More recently, SSW-OCTA employed a spatially weighted window mechanism to address capillary complexity. However, its high computational demand and the potential for smoothing out low-contrast capillary edges limit its efficiency and precision in subtle capillary recovery.

Current methods still face distinct technical limitations. Standard U-Net variants rely on local convolutions, which fail to capture the long-range spatial dependencies necessary for maintaining capillary continuity across the entire scan. Conversely, pure Transformer-based models such as Swin-Unet treat images as sequences, which often leads to a loss of fine-grained localization information, resulting in ‘blurry’ or over-smoothed capillary boundaries. Whereas hybrid models such as TransUNet attempt to combine both strengths, they frequently struggle with multi-scale feature misalignment, where low-contrast capillary information is ‘washed out’ during the fusion of CNN and Transformer paths.

To bridge these gaps, these considerations motivate the development of an architecture that can dynamically focus on capillary regions while compensating for information loss in low-contrast zones. This motivates our proposed RCNet, which utilizes BRA to optimize global feature extraction, CBM to refine strongly correlated capillary details, and DSCM to ensure robust information flow in signal-weak regions.

## 3. Method

### 3.1. RCNet Architecture

**Figure 3:**
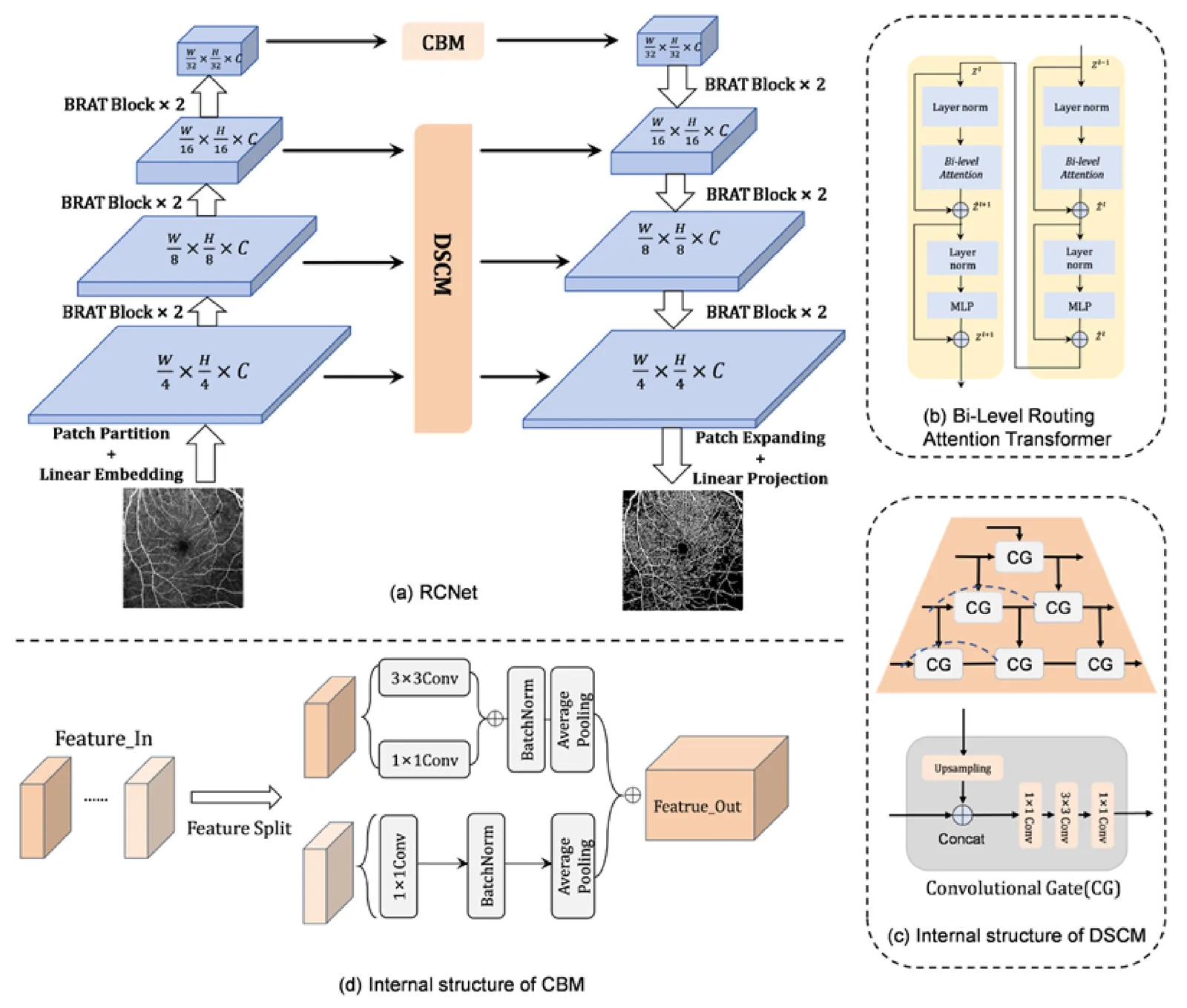
RCNet Overall Architecture.

The structure of RCNet is shown in Figure 3, consisting of BRAT, CBM, and DSC modules. First, the OCTA image is fed into the encoder module. To convert the image into a sequence embedding, the W × H capillary image is divided into 4 × 4 non-overlapping patches. The feature dimension is projected through a linear embedding layer, then feature maps are generated via the BRAT block and Patch Merging layer. The Patch Merging layer handles downsampling, while the BRAT block performs feature learning. After processing by the encoder, the feature map is fed into the CBM to compensate for the BRAT block’s limitations in extracting local detail features within the image. The decoder module consists of a BRAT block and a Patch Expanding layer. The Patch Expanding layer upsamples the CBM-processed feature map to a higher resolution by a factor of 2, ultimately restoring the feature map’s resolution to the input dimensions W × H. These upsampled feature maps then undergo Linear Projection to output pixel-level segmentation predictions, completing the retinal capillary image segmentation task. Additionally, we incorporate DSCM between the decoder and encoder to fuse extracted contextual features with multi-scale features from the encoder, mitigating spatial information loss caused by downsampling.

The RCNet model training process is shown in Algorithm 1.
**Algorithm 1: The training procedure of RCNet**Input: Images S = {x_i_, i ∈ ℕ}, Masks $T=\left\{y_{i}^{t},\;\mathrm{i\epsilon}N\right\}$
Output: Model parameters **for** i = 0 → batch size **do**   x = Patch Embedding(x_i_)   **for** m = 0 → stage_num_ **do**      **for** n = 0 → stage_num__BRAT **do**         x = x + pos_embed(x)         x = x + Bi − level Routing Attention(x)         x = x + MLP(x)      **end**      x_m_ = Patch Merging(x)      temp_m_ = x_m_   **end**   x = CBM(x)   **for** i = stage_num_ − 2 → −1 **do**      x_i_ = Patch Expanding(x)      x = Concat(temp_i_,x_2−i_)      x = DSCM(x)      **for** j = 0 → stage_num__BRAT **do**         x = x + pos_embed(x)         x = x + Bi − level Routing Attention(x)         x = x + MLP(x)      **end**   **end**   x = Patch Expanding(x)   $y_{i}^{\mathrm{out}}=\mathrm{Linear}\;\mathrm{Projection}(x)$
   Calculating the loss   Gradient back propagation, update parameters **end**

### 3.2. Bi-Level Routing Attention Transformer

Traditional CNNs primarily rely on local convolution operations, limiting their ability to capture long-range dependencies. In contrast, the Swin Transformer [32] leverages self-attention mechanisms to capture long-term dependencies within input sequences. This enables it to better understand and integrate relationships between distant pixels, enhancing precise segmentation in complex image scenes. However, when the Transformer computes pairwise token interactions across all spatial positions, model performance degrades significantly. Second, traditional attention mechanisms exhibit high computational time complexity, slowing inference speeds. Computational burden increases substantially with higher input resolutions, particularly for Multi-Head Attention, whose complexity rises sharply with input size. Furthermore, their limitations in modeling local details pose challenges when capturing fine features, especially when processing high-resolution images.

Given that capillary images are locally dense, this method employs a two-stage routing attention mechanism [33] as the Transformer’s attention mechanism. Compared to sparse capillary regions, this approach better focuses attention on densely vascularized areas for feature extraction, addressing the coarse granularity issue in traditional Transformer models when segmenting fine features in OCTA images. The processing steps are as follows:

(1) Region Partition and Linear Projection. Feature map X ∈ ℝ^H×W×C^ is divided into S × S disjoint regions such that each region contains $\frac{H\times W}{S^{2}}$ feature vectors. Convert it to $X^{r}\in R^{S^{2}\times \frac{H\times W}{S^{2}}\times C}$, then can obtain ${Q,K,V\in R}^{S^{2}\times \frac{H\times W}{S^{2}}\times C}$, by linear projection:
  (1) Q = X^r^W^q^,K = X^r^W^k^,V = X^r^W^v^
  W^q^, W^k^, W^v^ are the linear projection weight matrices for query, key, and value, respectively.
(2) Region-to-Region Routing. To identify the participating regions by constructing a directed graph, first calculate the average values of Q and K for each region to obtain ${Q^{r},K}^{r}\in R^{S^{2}\times C}$, then by applying matrix multiplication between Qr and Kr, we obtain the region-to-region adjacency matrix $A^{r}\in R^{S^{2}\times S^{2}}$. Finally, by using the routing index matrix $I^{r}\in N^{S^{2}\times K}$, the top K most relevant regions are reserved for each query region. These regions are then treated as relevant regions and participate in fine-grained operations. Region-to-region routing can be expressed as:
  (2) $$A^{r}=Q^{r}{\left(K^{r}\right)}^{T}$$
  (3) I^r^ = topkIndex(A^r^)
(3) Token-to-Token Attention. The most relevant regions are used as keys and values in the final computation. Since routing regions may be scattered across the entire feature map, it is necessary to collect the key and value tensors within these routing regions. Subsequently, fine-grained token-to-token attention is applied to these key-value tensors, which can be expressed as:
  (4) K^g^ = gather(K,I^r^),V^g^ = gather(V,I^r^)
  (5) $$O=\mathrm{softmax}\left(\frac{Q{\left(K^{g}\right)}^{T}}{\sqrt{C}}\right)V^{g}+\mathrm{LCE}\left(V\right)$$
  ${K^{g},V}^{g}\in R^{\mathrm{kHW}\times C}$ are the key tensor and value tensor, respectively. To enhance locality, deep convolutions are employed for parameterization.

**Figure 4:**
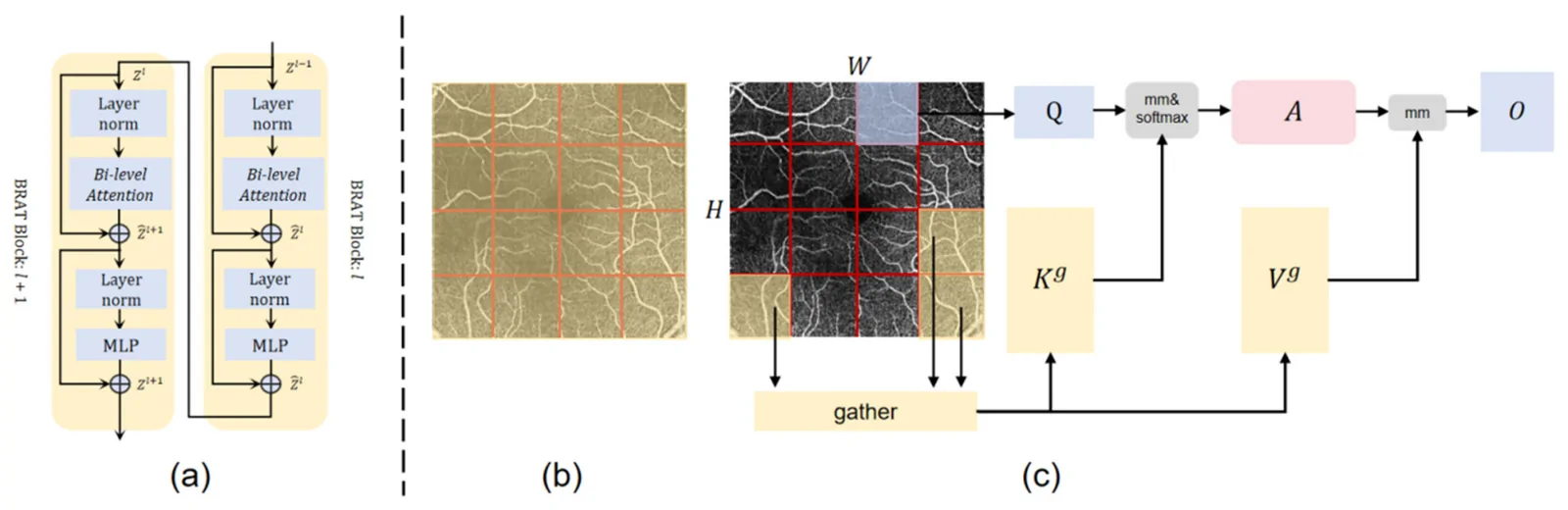
The position of attention within BRAT is illustrated in (**a**), where ${\hat{Z}}^{l}$ denotes the output of Bi-Level Routing Attention, Z^l^ represents the output of the MLP, and l indicates the block position. (**b**) shows the original attention implementation, which operates directly on a global scale, resulting in increased computational complexity and substantial memory consumption. The mechanism of bi-level routing attention is illustrated in (**c**).

The mechanism of bi-level routing attention is illustrated in Figure 4c. It filters out partially irrelevant key-value pairs while retaining key dense regions. These dense capillary areas are then prioritized for fine-grained computations and attention interactions. By restricting attention to selected regions, this mechanism focuses feature interactions on potentially relevant vascular areas; its effect on end-to-end inference efficiency cannot be inferred from the attention design alone.

In the proposed method, set S = 8 for the 256 × 256 input. The choice of S is based on the downsampling rate of the encoder. Because the feature map at the deepest stage is 8 × 8, setting S to match this resolution ensures that the “coarse-grained” routing grid aligns perfectly with the feature map boundaries, avoiding information misalignment and extra padding. The K value defines the sparsity of the attention. We followed a progressive strategy across the four stages of the encoder, setting K = [1, 4, 16, S^2^]. In the earlier stages, a smaller K was used to filter out background noise in the OCTA images, whereas in deeper stages, K was increased to capture more complex, multi-scale capillary textures.

### 3.3. Hybrid Network Architecture for Image Semantic Segmentation

Pure convolutional networks, pure Transformer networks, or hybrid networks with modified encoder–decoder architectures all yield mediocre results for OCTA capillary segmentation. However, it is undeniable that U-shaped architectures hold advantages for medical image segmentation. Therefore, we focus our attention on skip connection layers and Bottleneck layers.

**Dense Skip Connection Module (DSCM)**. The skip connection within the Transformer resembles the residual connection in ResNet [34]. Inspired by this, the skip connection between the Encoder and Decoder is replaced with a Dense Skip Connection. By stacking consecutive convolutions to build deep networks, this architecture facilitates capturing richer feature representations. The feature extraction capability of DSCM, combined with the residual connection concept from ResNet within the Transformer module, enhances the model’s deep understanding of data. It provides more effective information transfer and feature fusion between the Encoder and Decoder.

The skip connections employed in U-Net or other models directly feed high-resolution feature maps from the encoder to the decoder. This results in the fusion of semantically dissimilar feature maps, leading to a loss of semantic information between encoder and decoder feature maps. Consequently, image segmentation accuracy is reduced, which is one of the pain points of such models. Unlike the skip connections employed in U-Net, this method utilizes dense skip connections [35] to enhance the original skip connection architecture. This approach aligns the semantic levels of encoder feature maps more closely with those of decoder feature maps, effectively bridging the semantic gap between encoder and decoder. Consequently, decoders can access encoder features that have undergone aggregated semantic scaling. X^i,j^ denotes the output, where i represents the index of the downsampling layer along the encoding direction and j represents the index of the dense convolutional layer along the skip path. The function H(∙) is a convolutional operation, μ(∙) denotes the upsampling layer. The feature map is computed as follows:

(6) $$X^{i,j}=\left\{\begin{array}{c} \;\;\;\;\;\;\;\;\;H(X^{i-1,y}),\;j=0\\ H(\left[{\left[X^{i,k}\right]}_{k=0}^{j-1},\mu \left(X^{i+1,j-1}\right)\right]),\;j>0 \end{array}\right.$$

Overall, DSCM compensates for the loss of image feature detail inherent in pure Transformer architecture while capturing features at different levels and perceptual fields of varying sizes. This approach yields richer multi-scale information, minimizing spatial information loss caused by the downsampling process.

**CNN Bottleneck Module (CBM)**. This method constructs a Convolutional-based Bottleneck module to fully extract features, addressing BRAT’s limitation in capturing locally dense capillary features. CBM is expressed as shown in Equation (7):

(7) $$\left[\begin{array}{c} y_{1}\\ \vdots \\ y_{m} \end{array}\right]=\left[\begin{array}{ccc} W_{11} & \cdots & W_{1,\mathrm{\alpha L}}\\ \vdots & \ddots & \vdots \\ W_{M,1} & \cdots & W_{M,\mathrm{\alpha L}} \end{array}\right]\left[\begin{array}{c} x_{1}\\ \vdots \\ x_{\mathrm{\alpha L}} \end{array}\right]+\left[\begin{array}{ccc} w_{1,\mathrm{\alpha L}+1} & \cdots & w_{1,L}\\ \vdots & \ddots & \vdots \\ w_{M,\mathrm{\alpha L}+1} & \cdots & w_{M,L} \end{array}\right]\left[\begin{array}{c} x_{\mathrm{\alpha L}+1}\\ \vdots \\ x_{L} \end{array}\right]$$

W_i,j_,j ∈ [1, αL] denotes the convolution kernel parameter on the high-capacity branch. w_i,j_,j ∈ [αL + 1, L] denotes the convolution kernel parameters on the lightweight branch. In this work, α was set to 0.5 as a fixed hyperparameter. At the bottleneck stage, the fixed split ratio allocates equal channel widths to the two computational branches; this design does not, by itself, establish an end-to-end inference-efficiency advantage. The partition is fixed according to channel index and is not determined by an explicit correlation score, channel-ranking operation, or input-dependent selection procedure. Therefore, the two groups represent computational branches rather than strongly and weakly correlated feature components. In this study, α was fixed at 0.5 before training to allocate equal channel widths to the two branches. This parameter controls the branch width and is not interpreted as a correlation threshold. Because no systematic hyperparameter sweep was conducted, no claim regarding the sensitivity of RCNet to α is made. The channel-split ratio α was fixed at 0.5 in the present experiments. Its influence on the balance between segmentation accuracy and computational cost was not systematically investigated and should be evaluated in future work. Additionally, this method employs the binary cross-entropy (BCE) loss function because of its effectiveness in pixel-wise classification tasks. OCTA image segmentation is challenging owing to low contrast, blurred boundaries between capillaries and background, and complex capillary structures. The BCE loss measures the discrepancy between predicted probabilities and ground-truth labels at the pixel level, which helps the model learn discriminative features for vessel segmentation. Notably, BCE does not explicitly address class imbalance; however, in our setting, it provides stable optimization and satisfactory performance. Therefore, it is adopted as a simple and effective loss function in this work.

(8) $$L_{\mathrm{BCE}}=-\frac{1}{N}\sum_{i=1}^{N}\left[y_{i}\log\left({\hat{y}}_{i}+\in \right)+(1-y_{i})\log(1-{\hat{y}}_{i}+\in )\right]$$

## 4. Experiments

### 4.1. Datasets and Settings

**Figure 5:**
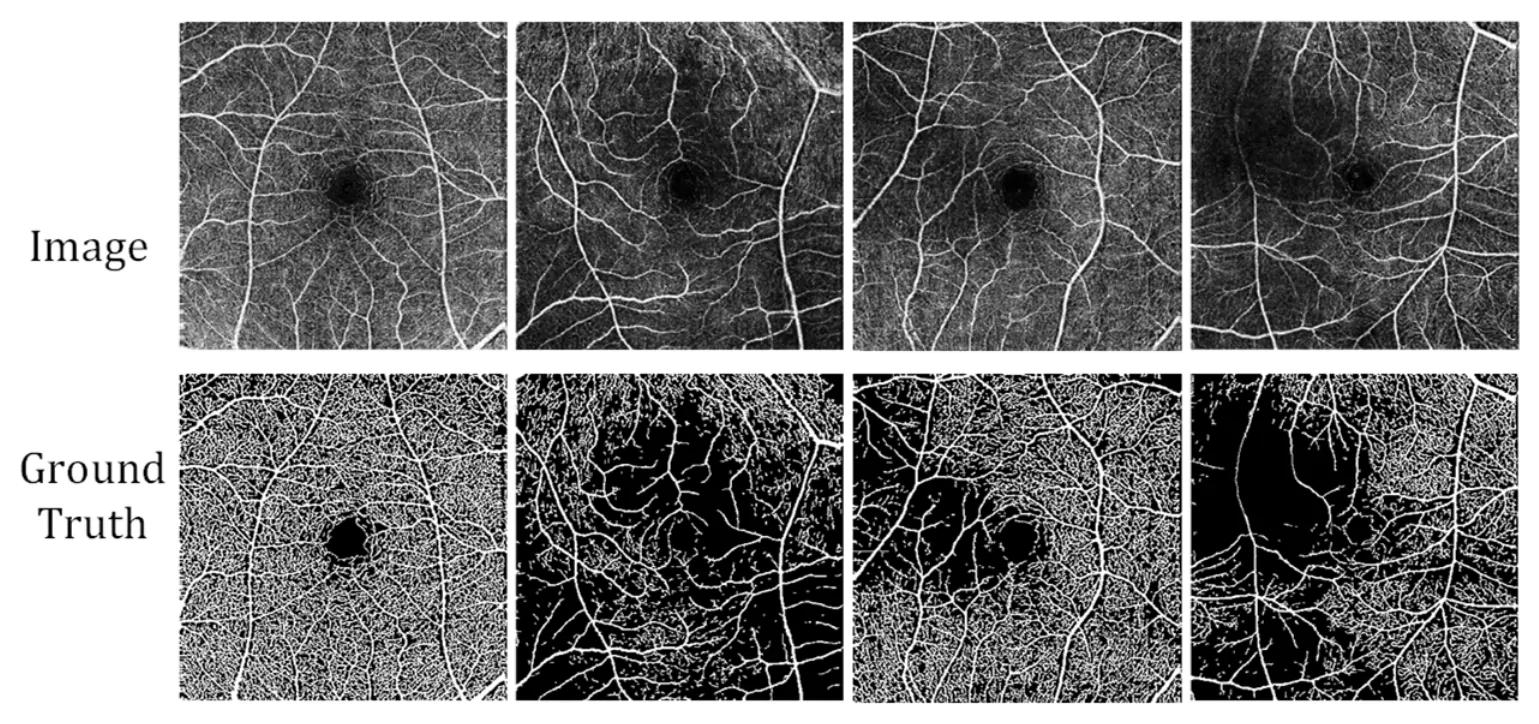
Grayscale images stored in 3-channel format and their corresponding binary labels at resolution size 400 × 400.

This study employs the publicly available OCTA-6M image dataset OCTA-500 for experimentation. The corresponding sample images are shown in Figure 5. The dataset provides high-resolution views of the retinal capillary system. The Image demonstrates morphological details of the retina, including the distribution and orientation of capillaries. Meanwhile, Ground Truth provides a reference standard for algorithmic learning by precisely annotating capillary contours, ensuring that the features learned by the model can be effectively applied to segmentation tasks.

The original dataset consisting of 300 images was first divided into training, validation, and test sets with a ratio of 6:2:2 (i.e., 180, 60, and 60 images, respectively). Data augmentation techniques, including flipping and rotation, were applied only to the training set to enhance model generalization, expanding it from 180 to 540 images. The validation and test sets remained unchanged during this process to ensure a fair evaluation. The RCNet network model was constructed using PyTorch 1.13.0 and trained on an NVIDIA GeForce RTX TITAN 24G GPU. We set each attention head to 32 channels. For BRA, we use *topk* = 1,4,16,S^2^ for the four stages, and the region partition factor S = 8. For CBM, α was set to 0.5. The batch size was set to 12, the learning rate to 0.0001, and the number of epochs to 130. The Adam algorithm was employed to update the model’s gradients. All comparator models were independently reproduced by the authors using publicly available implementations associated with the original publications or established public implementations when official author code was unavailable. The reported comparator results were not copied from the corresponding papers. To ensure a controlled comparison, all models were trained using the same dataset partitions, input resolution, augmentation procedure, optimizer, learning rate, number of epochs, loss function, checkpoint-selection criterion, and evaluation pipeline as RCNet.

### 4.2. Evaluation Metrics

The model output logits were converted into foreground probabilities using the sigmoid function. A fixed threshold of 0.5 was then applied to generate the binary prediction masks. The same threshold was used for all compared methods. To evaluate the segmentation performance of the RCNet model, this paper combines four metrics: DSC (Dice Similarity Coefficient), Jaccard, Precision, and Recall. Given a predicted segmentation mask X and ground truth labels Y, |X| and |Y| denote the areas of the segmentation results and labels respectively. |X∩Y| represents the area of overlap between segmentation results and labels, while |X∪Y| indicates the total area covered by segmentation results and labels. The Dice Similarity Coefficient is used to calculate the similarity between two samples, specifically measuring the overlap between segmentation results and labels. It is more sensitive to both the internal regions of the segmentation area and the true regions. Its definition is as follows:

(9) $$\mathrm{DSC}=\frac{2\left|X\cap Y\right|}{\left|X\right|+\left|Y\right|}$$

To further evaluate the topological fidelity of segmented vessel structures, this study additionally employed clDice [36] (centerline Dice), a metric specifically designed to assess the alignment between predicted and ground-truth centerlines—crucial for preserving connectivity and branching patterns in tubular structures such as capillaries. Let S_X_ and S_Y_ denote their respective skeletal representations (i.e., centerlines extracted via morphological thinning or skeletonization). The clDice coefficient measures the overlap between these centerlines while also considering the original segmentation masks to ensure spatial consistency. Its definition is as follows:

(10) $$\mathrm{clDice}=\frac{2\left|S_{X}\cap Y\right|\left|S_{Y}\cap X\right|}{\left|S_{X}\cap Y\right|\left|S_{Y}\right|+\left|S_{Y}\cap X\right|\left|S_{X}\right|}$$

For clDice calculation, the predicted and ground-truth binary masks were skeletonized independently using the two-dimensional Zhang–Suen thinning algorithm implemented by skimage.morphology.skeletonize. Topological precision was calculated by measuring the overlap between the predicted skeleton and the ground-truth mask, while topological sensitivity was calculated by measuring the overlap between the ground-truth skeleton and the predicted mask.

The Jaccard index is used to compare the similarity between sample sets. It serves as a measure of the degree of overlap between two sets, defined as the ratio of the size of their intersection to the size of their union. Its definition is as follows:

(11) $$\mathrm{Jaccard}=\frac{\left|X\cap Y\right|}{\left|X\cup Y\right|}$$

Precision focuses on the accuracy of model predictions. A higher value indicates a greater likelihood that the model correctly predicts all positive instances within a class. Models with high precision rarely misclassify negative instances as positive. It is defined as follows:

(12) $$\mathrm{Precision}=\frac{\left|X\cap Y\right|}{\left|X\right|}$$

Recall is another commonly used metric in the field of deep learning, serving to quantify the comprehensiveness of a model’s predictions. A model with high recall can identify the majority of positive class samples. Its definition is as follows:

(13) $$\mathrm{Recall}=\frac{\left|X\cap Y\right|}{\left|Y\right|}$$

### 4.3. Comparison with Typical Segmentation Models

To validate the model’s performance, this paper selected multiple state-of-the-art deep learning models as comparison methods for RCNet. These models include U-Net, DeepLabV3+ [37], Swin-Unet, TransUNet, SegFormer [38], Mask2Former [39], R2U-Net [40], HiFormer [41], UNext [42], OCTA-Net [27] and SSW-OCTA [43]. These models were chosen due to their unique advantages and extensive application bases in the field of image segmentation. U-Net is a classic medical image segmentation model. Its symmetric encoder–decoder architecture and feature fusion strategy enable it to capture contextual information within images while preserving edge details. DeepLabV3+ employs multi-scale feature extraction and ASPP modules to ensure precise segmentation in complex scenarios involving both semantic and instance segmentation. Swin-Unet is a network built upon the Swin Transformer, leveraging the Transformer’s global self-attention mechanism to capture long-range dependencies across images. TransUNet enhances medical image segmentation performance through a hybrid CNN-Transformer architecture. SegFormer ensures high-performance segmentation while optimizing computational cost via a hierarchical Transformer encoder and lightweight MLP decoder. Mask2Former employs an innovative object query design to enhance the model’s focus on distinct regional features. HiFormer and UNeXt improve the efficiency and performance of Transformers in processing high-resolution medical images through novel network designs for medical image segmentation tasks. HiFormer utilizes a hierarchical structure and multi-scale attention mechanism, effectively combining local and global attention to achieve precise modeling of fine-grained features and long-range dependencies. UNeXt integrates an efficient Tokenized MLP module into the classic U-Net architecture, replacing computationally intensive self-attention mechanisms while retaining shallow CNNs for local feature extraction. OCTA-Net is a split-based coarse-to-fine capillary segmentation network specifically designed for OCTA images that detects thick and thin vessels separately through a two-stage module that first generates a preliminary confidence map and then refines the shape and contour of retinal capillaries. SSW-OCTA integrates deformable convolutions suited for tubular structures with the Swin Transformer for global feature extraction, adapting to the characteristics of OCTA modality images to achieve robust retinal capillary segmentation. This approach maintains competitive segmentation performance while significantly reducing model parameters and computational overhead, making it particularly suitable for scenarios demanding high efficiency. By comparing these advanced models, we can evaluate RCNet’s performance when processing OCTA retinal capillary images. The comparative experimental results are shown in Table 1.

The comprehensive experimental results on the OCTA-500 dataset are summarized in Table 1. As indicated by the data, the proposed RCNet achieved the highest scores across several primary evaluation metrics, including a DSC of 60.47%, a clDice of 59.39%, and a Jaccard score of 43.68%. These results indicate favorable performance in preserving capillary regions and topological connectivity under the evaluated setting.

A critical distinction can be observed between RCNet and other specialized capillary segmentation models such as OCTA-Net and SSW-OCTA. While OCTA-Net is a well-regarded two-stage framework, it yields a DSC of 60.03% and a clDice of 57.61%. RCNet surpasses it by 1.78% in clDice, suggesting a stronger ability to maintain the continuity of fine capillaries. Furthermore, although SSW-OCTA achieves a competitive Jaccard index of 43.11%, it incurs a significant computational penalty with 116.44 M parameters and 192.27 G FLOPs. In comparison, RCNet attains better segmentation accuracy with 43.56 M parameters and 56.96 G FLOPs.

Regarding foundational benchmarks, RCNet provides a consistent lead over the classic U-Net, improving the DSC and Jaccard scores by 1.56% and 1.58% respectively. General-purpose models such as DeepLabV3+, SegFormer, and Mask2Former exhibit lower performance on this specialized dataset, which highlights the difficulty of applying standard multi-scale or mask-prediction mechanisms to the sparse and intricate structures of the retinal microvasculature.

In terms of the Recall metric, TransUNet achieves the highest score of 52.12%. However, its Precision remains notably low at 59.81%, whereas RCNet maintains a much higher Precision of 75.73%. This indicates that while TransUNet may recover more capillary pixels, it suffers from a higher rate of false-positive detections. Other hybrid models like UNeXt and HiFormer optimize for model size but fall short of RCNet in terms of capillary connectivity. Overall, the superior performance of RCNet across balanced metrics supports the effectiveness of the feature interaction design under the evaluated settings at capturing complex capillary details.

The experimental findings validate the limitations of pure Transformer models in extracting detailed features from medical images. By enhancing the attention mechanism of Transformer blocks and incorporating convolutional-based DSCM and CBM, the RCNet effectively fuses multi-scale features, thereby improving segmentation performance. This demonstrates the superiority of the CNN-Transformer hybrid architecture for segmenting OCTA retinal capillary images. To facilitate comparison and analysis of segmentation results across models, we visualized the segmentation outputs for each model.

**Figure 6:**
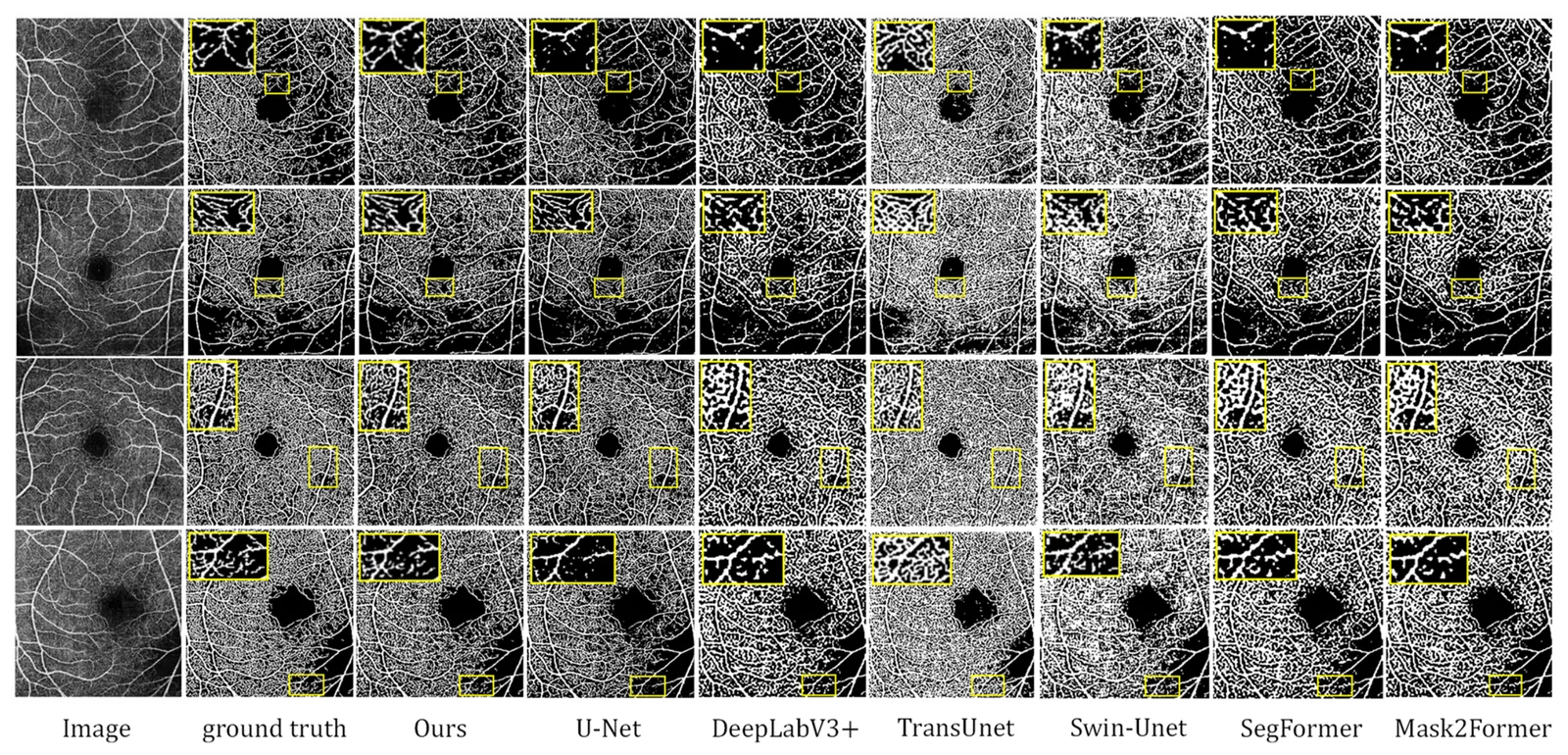
Comparison of segmentation performance across different neural network models on the OCTA-500 dataset.

As shown in Figure 6, we enlarged local image details for intuitive comparison. Both the details within the box and the entire image reveal that while the U-Net model achieves good segmentation in dense capillary regions, its weaker ability to capture long-range spatial dependencies leads to missed capillaries in sparse areas. For TransUNet, despite combining the strengths of CNNs and Transformers, overfitting is prone to occur. Regarding the Swin-Unet model, while the pure Transformer architecture excels at modeling global information, it struggles with capillary structures. By treating inputs as one-dimensional sequences, it lacks detailed localization information for low-resolution features, preventing effective recovery of fine-grained details and resulting in coarse segmentation outcomes.

RCNet’s prediction maps are significantly clearer than those of other models. For images with dense and complex capillary structures, our model produces more continuous and detailed segmentation results. Compared to Transformer-based models like Mask2Former and SegFormer, our approach achieves superior performance in fine-grained segmentation, enabling the extraction of more intricate capillary branches and ensuring high precision in capillary segmentation. Compared to convolutional network-based models like U-Net and DeepLabV3+, our approach maintains segmentation continuity for sparse capillaries.

Notably, while some models slightly outperform our method in certain metrics, their visual segmentation quality falls short. This discrepancy arises because area-oriented metrics like Dice/IoU exhibit varying sensitivity to different types of segmentation errors. Competitive models prioritize large-area trunk vessels through conservative predictions to maximize overall metrics, sacrificing detection of capillaries. While our approach may introduce minor false positives at boundaries, it better preserves capillary network topology and connectivity.

To further validate the robustness and generalization capabilities of RCNet, we conducted comprehensive experiments on the ROSE dataset. This dataset, comprising 229 clinical OCTA images, was partitioned into training, validation, and testing sets according to a 6:2:2 ratio. As presented in Table 2, RCNet achieved superior performance across all evaluation metrics, yielding a DSC of 84.41%, a clDice of 80.21%, and a Jaccard index of 73.41%. RCNet also attained the highest Precision and Recall among all compared methods, which underscores its balanced capability in both pixel-wise accuracy and vascular detection.

The advantages of RCNet are particularly evident compared to specialized capillary segmentation frameworks such as OCTA-Net and SSW-OCTA. Although OCTA-Net demonstrates strong performance with a DSC of 82.02%, its clDice score is limited to 75.65%. RCNet surpasses OCTA-Net by 4.56% in clDice, representing an observed improvement in preserving the topological connectivity of intricate retinal capillaries. Furthermore, whereas SSW-OCTA is designed for complex capillary modeling, it achieves a lower DSC of 74.52% on this dataset. From a computational perspective, SSW-OCTA is considerably heavier, requiring 116.44 M parameters and 97.23 G FLOPs. On ROSE, RCNet achieved higher segmentation scores than SSW-OCTA while using fewer parameters and FLOPs in the evaluated configurations. These differences characterize segmentation performance and nominal model complexity.

In comparison with foundational architectures, RCNet outperforms the classic U-Net by 2.46% in DSC and 2.16% in clDice, suggesting that the proposed feature interaction mechanism is more effective than standard skip-connection strategies. Transformer-based models such as SegFormer and Mask2Former exhibit performance bottlenecks on the ROSE dataset, with DSC scores of 71.41% and 71.74% respectively. This suggests that pure Transformer architectures may struggle to localize the fine-grained boundaries of capillaries without specialized spatial priors.

Within the hybrid category, while TransUNet, HiFormer, and UNeXt leverage various fusion mechanisms, they fall short of RCNet in capturing capillary details and connectivity. For instance, although UNeXt is the most lightweight model in the comparison, its DSC is limited to 66.81%. The overall performance gap highlights that RCNet effectively mitigates the loss of spatial information during downsampling. The consistent competitive performance of RCNet on the ROSE dataset demonstrates its potential as a promising approach for retinal capillary segmentation under the evaluated conditions for high-precision segmentation of retinal capillaries.

**Figure 7:**
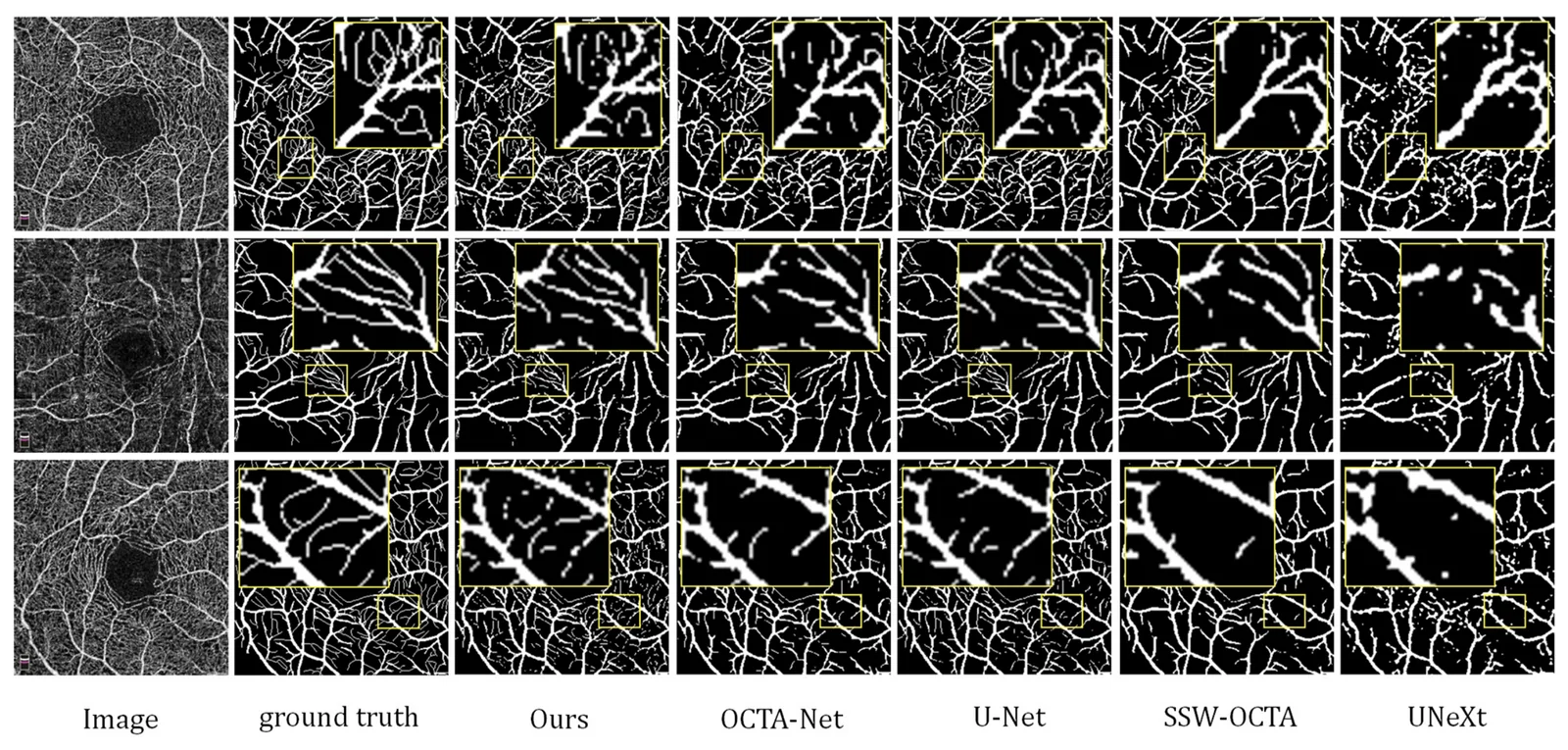
Comparison of segmentation performance across different neural network models on the ROSE dataset.

To qualitatively evaluate the segmentation performance, we present a visual comparison between RCNet and several baseline models on the ROSE dataset in Figure 7. As illustrated in the enlarged yellow bounding boxes, RCNet demonstrates a distinct advantage in preserving the topological connectivity of capillaries. Our model effectively recovers capillary segments that are often fragmented or omitted by other methods, maintaining a good degree of fidelity to the ground truth.

Although U-Net and OCTA-Net achieve relatively better results on larger vessel structures, they exhibit noticeable deficiencies when processing delicate capillary tips and complex bifurcations. In these challenging areas, these two models frequently produce over-smoothed capillary boundaries or broken branches, which limits their ability to accurately represent the terminal ends of the capillary network. By contrast, SSW-OCTA and UNeXt suffer from more severe capillary discontinuities and prominent speckle artifacts throughout the dense capillary regions. These artifacts and breaks lead to a cluttered segmentation mask that fails to represent the continuous nature of the retinal capillaries.

The superior capillary segmentation results of RCNet are highly consistent with the quantitative metrics reported in Table 2. Specifically, the visual integrity of the microvascular network corresponds to high clDice scores, whereas the reduction in noise and precise boundary localization align with the superior DSC and Precision metrics. These qualitative observations are consistent with the quantitative results and suggest that RCNet preserves vascular morphology more effectively in the displayed examples by capturing the essential morphological details and morphological integrity required for the high-fidelity assessment of retinal capillaries.

### 4.4. Ablation Study

**Figure 8:**
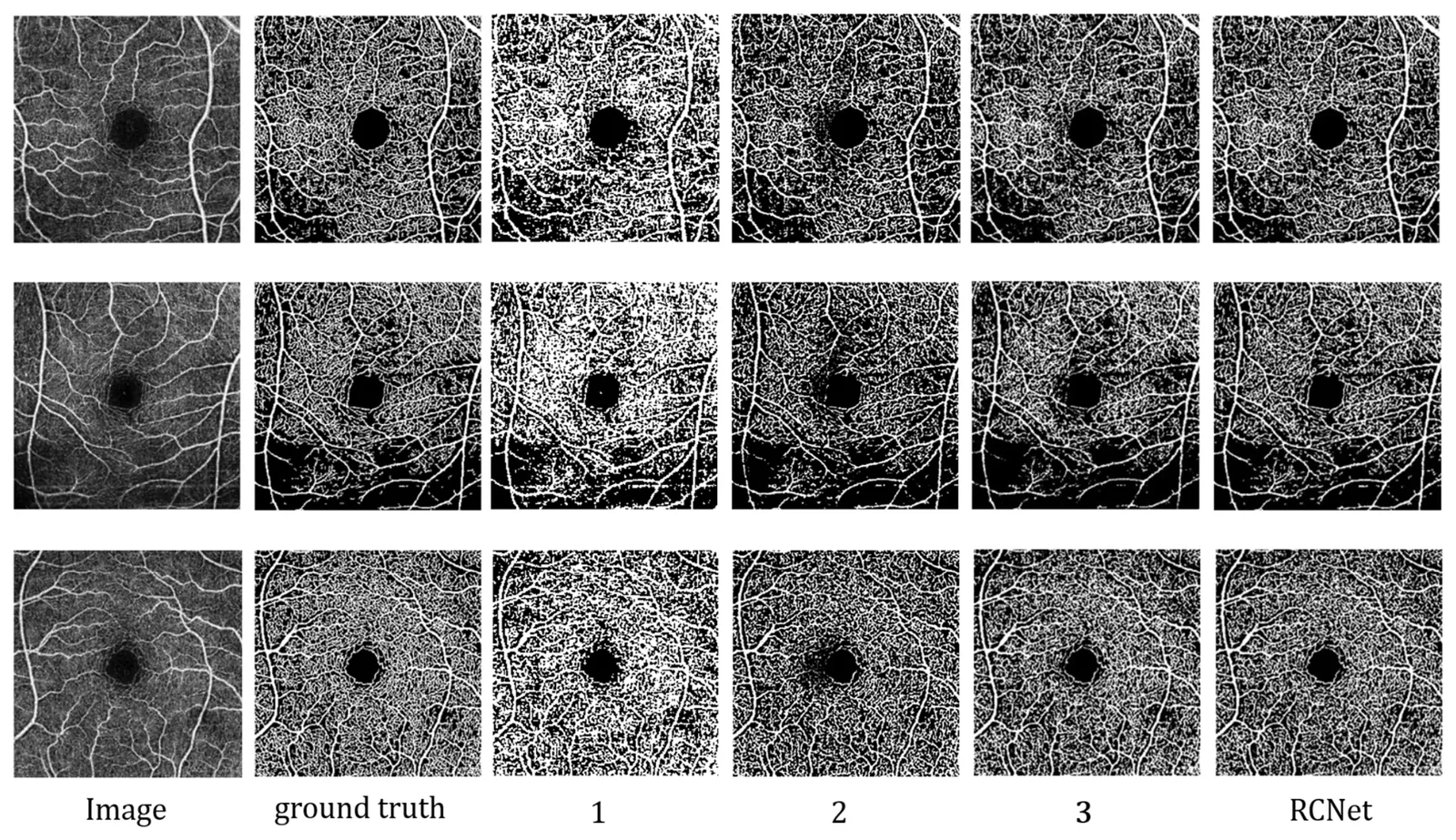
Visual comparison of the impact of each module on network performance.

To assess both the individual contributions and the complementary effects of BRAT, CBM, and DSCM, we conducted a systematic ablation study involving eight architectural configurations on the OCTA-500 dataset. As shown in Table 3, the baseline Transformer-based encoder–decoder achieved a DSC of 56.75% and a Jaccard index of 39.97%, indicating that the baseline alone has limited ability to recover the fine and complex structures of the capillary network. When introduced individually, BRAT produced the largest improvement, increasing the DSC to 58.48% and the Jaccard index to 41.32%. This result suggests that bi-level routing attention facilitates the modeling of spatially distributed capillary regions and long-range vascular dependencies. DSCM alone increased the DSC and Jaccard index to 58.19% and 41.03%, respectively, indicating that dense feature transfer between the encoder and decoder helps preserve contextual and spatial information. CBM alone achieved a DSC of 57.30% and a Jaccard index of 40.15%. Although its individual improvement was relatively modest, the result indicates that additional convolutional processing at the bottleneck contributes to local feature refinement. The experiments with different module combinations further demonstrate their complementary roles. Among the two-module configurations, BRAT + DSCM achieved the best performance, with a DSC of 59.60% and a Jaccard index of 42.45%, suggesting that global dependency modeling and multi-level feature transfer are particularly complementary for capillary segmentation. BRAT + CBM achieved a DSC of 59.03% and a Jaccard index of 41.87%, while CBM + DSCM achieved 58.52% and 41.36%, respectively. These results also show that each two-module configuration outperformed its corresponding single-module variants. Finally, integrating BRAT, CBM, and DSCM into the complete RCNet further increased the DSC to 60.47% and the Jaccard index to 43.68%, representing improvements of 3.72 and 3.71 percentage points over the baseline, respectively. Compared with the strongest two-module configuration, the complete model provided additional improvements of 0.87 percentage points in DSC and 1.23 percentage points in Jaccard. Overall, the results indicate that BRAT provides the principal improvement in global feature modeling, DSCM supports the preservation and transfer of multi-level spatial information, and CBM supplies complementary local refinement. Their integration enables RCNet to achieve the best overall performance among the evaluated configurations. The corresponding qualitative results are presented in Figure 8.

Ablation experiments reveal that all three improvements positively impact the model’s segmentation results. The model begins capturing finer capillary structures, with segmentation accuracy gradually increasing. This enables the hybrid CNN-Transformer encoder–decoder architecture to better perform capillary image segmentation tasks.

### 4.5. Comparison of Loss Functions

To investigate whether an imbalance-aware objective could improve capillary segmentation, we further compared the original binary cross-entropy (BCE) loss with focal loss. Focal loss reduces the contribution of easily classified pixels and places greater emphasis on difficult samples. The focusing parameter gamma and balancing parameter alpha were set to 2 and 0.25, respectively. For a controlled comparison, only the loss function was changed, while the network architecture, dataset partition, data augmentation, input resolution, optimizer, learning rate, batch size, number of training epochs, checkpoint-selection criterion, and prediction threshold remained unchanged.

As shown in Table 4, focal loss increased Recall from 51.66% to 52.40%, indicating that emphasizing difficult pixels enabled the model to recover additional capillary pixels. However, Precision decreased from 75.73% to 71.85%, suggesting that some low-contrast background regions and ambiguous boundaries were also incorrectly identified as capillaries. Consequently, focal loss produced lower DSC, clDice, and Jaccard scores of 59.26%, 58.42%, and 42.11%, respectively, compared with 60.47%, 59.39%, and 43.68% obtained using BCE.

These results indicate that the increased sensitivity of focal loss was accompanied by a higher false-positive rate. Under the present experimental setting, BCE provided a more favorable balance between capillary recovery and false-positive suppression and was therefore retained as the training objective for the primary RCNet experiments. Moreover, because all architecture-level comparisons in Table 1 and Table 2 were conducted using the same BCE objective, the observed performance improvements can be attributed primarily to the proposed architectural design rather than to the use of a specialized imbalance-aware loss function.

### 4.6. Statistical Analysis

To quantify the uncertainty associated with the finite test set and enable paired comparisons on the same images, we performed a non-parametric paired bootstrap analysis of the image-level clDice scores on the 60 OCTA-500 test images. The analysis included RCNet and the two closest-performing comparator models in terms of clDice, OCTA-Net and SSW-OCTA. For each of 10,000 bootstrap replicates, 60 test-image indices were sampled with replacement. The same sampled indices were applied to all three models within each replicate to preserve the paired correspondence of their predictions. The mean clDice was then calculated for each model, and the differences between RCNet and each comparator were calculated within the same replicate. The 95% confidence intervals were obtained from the 2.5th and 97.5th percentiles of the corresponding bootstrap distributions.

As reported in Table 5, RCNet achieved a mean clDice of 59.39% with a 95% confidence interval of 57.66–61.16%. The corresponding values were 57.61% (95% CI: 55.74–59.54%) for OCTA-Net and 57.15% (95% CI: 55.41–58.94%) for SSW-OCTA. In the paired comparisons, RCNet exceeded OCTA-Net by 1.78 percentage points (95% CI: 0.98–2.56) and SSW-OCTA by 2.24 percentage points (95% CI: 1.35–3.15). Neither paired confidence interval included zero, indicating that the clDice advantage of RCNet was consistently observed across bootstrap resamples of the test images. These results provide additional evidence that RCNet better preserves capillary topology and connectivity under the evaluated test setting. This bootstrap analysis characterizes uncertainty associated with test-set sampling and complements the point estimates reported in the primary experiments.

### 4.7. Limitations

**Figure 9:**
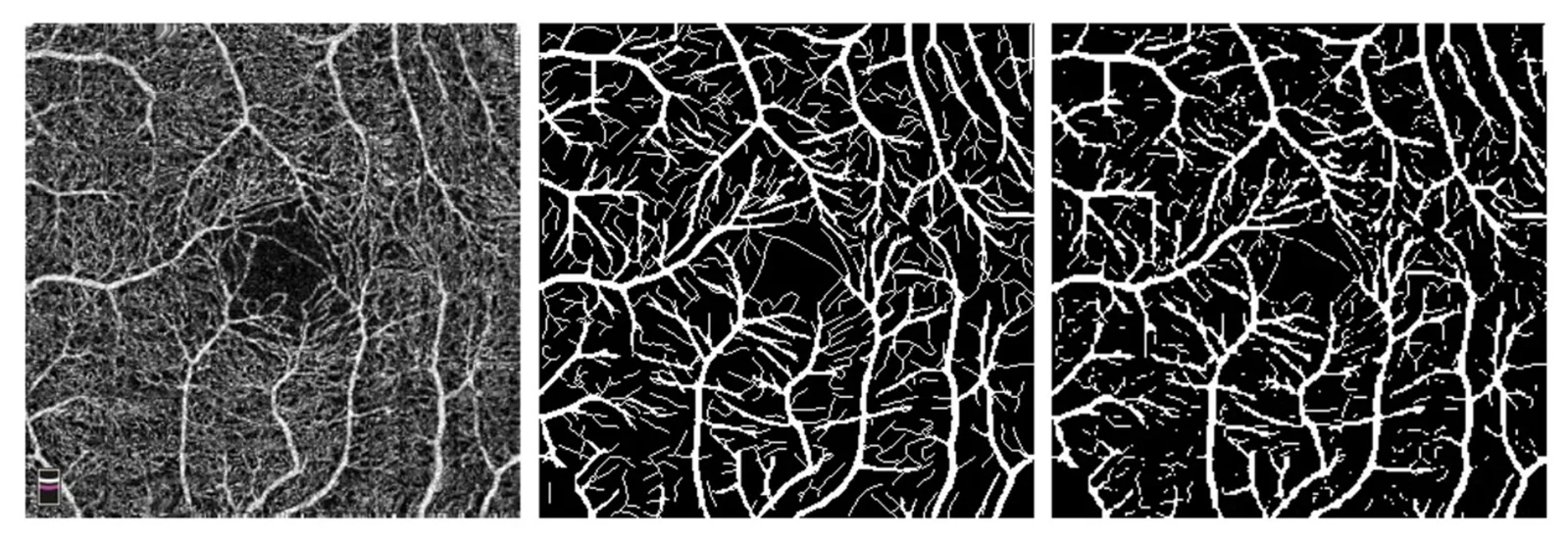
Failure cases of the proposed method in the ROSE dataset (left: original, middle: ground truth, right: prediction).

Despite the competitive performance achieved by the proposed method across most evaluation metrics and models, certain failure cases are observed in complex capillary regions, as exemplified in Figure 9. Specifically, in ROSE, the segmentation output exhibits incomplete delineation of ultra-fine capillary branches and topological discontinuities in areas characterized by high vascular density and intricate branching morphology. The predicted vascular mask demonstrates a tendency toward over-smoothing of thin-caliber vessels, resulting in fragmented skeletons and the loss of terminal capillary endpoints. These failure modes can be attributed to certain interrelated factors. The inherently low signal-to-noise ratio of capillaries in OCTA imagery leads to insufficient contrast between capillary walls and the surrounding background, making their feature representations easily suppressed during deep feature extraction. The class imbalance between thin capillary pixels and background causes the network’s loss function to under-penalize missed predictions on fine structures.

Several limitations remain that warrant further investigation. First, the scale and diversity of the datasets. Although RCNet was validated on two authoritative datasets, OCTA-500 and ROSE, the total number of clinical samples remains relatively limited compared to large-scale natural image datasets. The current model was primarily trained and tested on high-quality images with relatively consistent acquisition parameters. In real-world clinical settings, OCTA images are often subject to various motion artifacts, signal attenuation, and hardware-specific noise from different manufacturers. Future research should focus on incorporating multi-center data and exploring domain adaptation techniques to enhance the model’s robustness and generalization across diverse imaging conditions. Second, the dimensionality of the segmentation task. Current experiments were conducted on 2D en-face maximum intensity projection images, which is the standard practice in many clinical assessments. However, OCTA is inherently a 3D imaging modality containing depth-resolved information across different retinal layers. Segmenting capillaries directly in 3D space could provide more comprehensive morphological insights into vascular pathologies. Therefore, extending RCNet to a 3D or pseudo-3D framework to exploit volumetric spatial dependencies represents a promising future research direction. Finally, the integration of auxiliary clinical information. While RCNet focuses purely on image-based feature learning, clinical diagnosis often relies on a combination of imaging and non-imaging data. Incorporating these multi-modal clinical priors into the network architecture could further refine the segmentation results. Although parameter counts and FLOPs are reported, we did not conduct a comprehensive, standardized comparison of inference latency, throughput, peak GPU memory usage, and model size across all methods. Consequently, the practical inference efficiency and deployment suitability of RCNet remain unestablished. Future work should evaluate these properties under a unified benchmarking protocol and investigate implementation-level optimization.

The reported results were obtained under one fixed dataset partition and one training run for each architecture. Although this controlled setting ensures that all models are compared using identical training and test images, it does not fully characterize performance variation caused by random initialization or stochastic optimization. Future work should evaluate RCNet using repeated random seeds or repeated subject-level partitions when larger-scale computational validation is available.

## 5. Conclusions

Currently, there is a lack of deep learning models capable of accurately segmenting OCTA 2D en-face retinal capillary images. To achieve precise segmentation of retinal capillaries in OCTA images, this study proposes RCNet, a hybrid architecture that combines Transformer-based global modeling via BRAT with local feature extraction through convolutional layers. Addressing the challenges of low contrast, blurred vessel-background boundaries, and dense, complex capillary structures in capillary images, the bi-level routing attention mechanism is introduced in both the encoder and decoder of RCNet. This mechanism more effectively focuses attention on multiple densely vascularized regions within the image, optimizing the extraction of global features. By constructing a Convolutional Bottleneck Module (CBM) after decoding in the encoder and a Dense Skip Connection Module (DSCM) between the encoder and decoder, the method overcomes the poor feature extraction capability of pure Transformer models on locally dense capillaries. These integrations enable RCNet to accurately segment capillaries, addressing the low segmentation accuracy of traditional networks—such as pure convolutional networks, pure Transformer networks, and CNN-Transformer hybrid networks—when processing OCTA retinal capillary images. By enhancing segmentation accuracy, this approach provides high-quality morphological representations that could benefit downstream quantitative analysis. Such precision offers insights into clinical assessment of ocular diseases.

## Figures and Tables

**Table 1:** Accuracy comparison results between RCNet and other methods on the OCTA-500 dataset. (**↑** indicates that a higher value corresponds to better performance.)

	Method	Params	FLOPs	DSC (%) ↑	clDice (%) ↑	Jaccard (%) ↑	Precision (%) ↑	Recall (%) ↑
CNN-based	U-Net	34.53 M	147.30 G	58.91	57.23	42.10	73.85	46.62
	R2U-Net	39.09 M	142.50 G	56.56	55.75	39.78	72.87	45.27
	DeepLabV3+	40.35 M	44.82 G	53.68	51.98	37.04	68.06	43.31
	OCTA-Net	71.65 M	99.67 G	60.03	57.61	43.27	74.32	49.86
Transformer-based	Swin-Unet	54.34M	45.56G	56.75	54.57	39.97	61.06	46.55
	SegFormer	80.82 M	135.40 G	57.36	54.27	40.56	69.91	48.23
	Mask2Former	68.10 M	254.40 G	57.17	53.91	40.38	70.02	47.04
Hybrid	TransUNet	105.28 M	78.50 G	58.52	56.32	41.71	59.81	**52.12**
	HiFormer	50.33 M	82.16 G	58.27	54.96	41.46	70.54	47.94
	UNeXt	15.58 M	25.8 G	58.71	55.64	41.90	70.11	48.78
	SSW-OCTA	116.44 M	192.27 G	59.84	57.15	43.11	74.24	50.23
Proposed	RCNet	43.56 M	56.96 G	**60.47**	**59.39**	**43.68**	**75.73**	51.66

**Table 2:** Comparison of accuracy between RCNet and other methods on the ROSE dataset.

	Method	Params	FLOPs	DSC (%) ↑	clDice (%) ↑	Jaccard (%) ↑	Precision (%) ↑	Recall (%) ↑
CNN-based	U-Net	34.53 M	65.47 G	81.95	78.05	71.04	87.51	78.81
	R2U-Net	39.09 M	64.68 G	75.34	71.41	62.56	80.45	70.38
	DeepLabV3+	40.35 M	20.55 G	65.13	60.30	51.05	71.70	59.96
	OCTA-Net	71.65 M	44.30 G	82.02	75.65	69.59	87.88	76.79
Transformer-based	Swin-Unet	54.34M	25.36 G	72.35	70.29	56.80	78.67	71.05
	SegFormer	80.82 M	61.71 G	71.41	65.16	55.78	76.63	66.34
	Mask2Former	68.10 M	110.85 G	71.74	64.89	56.53	75.49	64.12
Hybrid	TransUNet	105.28 M	49.22 G	70.32	66.34	55.71	74.52	67.45
	HiFormer	50.33 M	52.67 G	71.25	67.92	58.46	75.09	66.74
	UNeXt	15.58 M	15.7 G	66.81	63.22	50.17	70.86	63.21
	SSW-OCTA	116.44 M	97.23 G	74.52	69.33	59.44	80.14	69.67
Proposed	RCNet	43.56 M	21.74 G	**84.41**	**80.21**	**73.41**	**88.32**	**80.83**

**Table 3:** Comparison of segmentation performance among ablation models.

Num	BRAT	CBM	DSCM	DSC (%)	Jaccard (%)
Baseline (RCNet 1)				56.75	39.97
Baseline + BRAT (RCNet 2)	✓			58.48	41.32
Baseline + DSCM			✓	58.19	41.03
Baseline + CBM		✓		57.30	40.15
Baseline + BRAT + DSCM	✓		✓	59.60	42.45
Baseline + CBM + DSCM		✓	✓	58.52	41.36
Baseline + BRAT + CBM (RCNet 3)	✓	✓		59.03	41.87
RCNet	✓	✓	✓	60.47	43.68

**Table 4:** Comparison of RCNet using different loss functions on the OCTA-500 dataset.

Loss Function	Alpha	Gamma	DSC (%) ↑	clDice (%) ↑	Jaccard (%) ↑	Precision (%) ↑	Recall (%) ↑
BCE	-	-	60.47	59.39	43.68	75.73	51.66
Focal Loss	0.25	2	59.26	58.42	42.11	71.85	52.40

**Table 5:** Image-level clDice performance and paired bootstrap comparisons on the OCTA-500 test set.

Model	clDice, % (95% CI)	RCNet − Method: clDice, Percentage Points (95% CI)
RCNet	59.39 (57.66, 61.16)	-
OCTA-Net	57.61 (55.74, 59.54)	+1.78 (+0.98, +2.56)
SSW-OCTA	57.15 (55.41, 58.94)	+2.24 (+1.35, +3.15)

## Data Availability

The data presented in this study are openly available in OCTA-500 at https://doi.org/10.1016/j.media.2024.103092, reference number [28], and in ROSE at https://doi.org/10.1109/TMI.2020.3042802, reference number [27]. Additional details on implementation sources, comparative training settings and bootstrap comparisons are provided in Supplementary Tables S1 and S2.

## Supplementary Materials

The following are available online at https://www.mdpi.com/article/10.3390/bioengineering13101147/s1, Table S1: Public implementation sources and unified training settings used for the comparative experiments. Table S2: Image-level performance and paired bootstrap comparisons on OCTA-500 test set.
