# Supplementary material for: RCNet: A Hybrid Network Adapted for OCTA Retinal Capillary Segmentation
Source: Bioengineering (Basel). 2026 Sep 30;13(10):1147. doi: 10.3390/bioengineering13101147 (PMC13701182; doi:10.3390/bioengineering13101147)
Supplement: Supplementary file 1 [file bioengineering-13-01147-s001.zip › Supplementary Table S1.pdf]

**Supplementary Table S1. Public implementation sources and unified training settings used for the comparative experiments.**

| Model | Public implementation source | Source type | Input size | Epochs | Batch size | Optimizer | Learning rate | Loss |
|---|---|---|---|---|---|---|---|---|
| U-Net | LeeJunHyun/Image_Segmentation | Public PyTorch implementation | 256 × 256 | 130 | 12 | Adam | $1 \times 10^{-4}$ | BCE |
| R2U-Net | LeeJunHyun/Image_Segmentation | Public PyTorch implementation | 256 × 256 | 130 | 12 | Adam | $1 \times 10^{-4}$ | BCE |
| DeepLabV3+ | tensorflow/models – DeepLab | Author-released implementation | 256 × 256 | 130 | 12 | Adam | $1 \times 10^{-4}$ | BCE |
| OCTA-Net | iMED-Lab/OCTA-Net | Official implementation | 256 × 256 | 130 | 12 | Adam | $1 \times 10^{-4}$ | BCE |
| Swin-Unet | HuCaoFighting/Swin-Unet | Official implementation | 256 × 256 | 130 | 12 | Adam | $1 \times 10^{-4}$ | BCE |
| SegFormer | NVlabs/SegFormer | Official implementation | 256 × 256 | 130 | 12 | Adam | $1 \times 10^{-4}$ | BCE |
| Mask2Former | facebookresearch/Mask2Former | Official implementation | 256 × 256 | 130 | 12 | Adam | $1 \times 10^{-4}$ | BCE |
| TransUNet | Beckschen/TransUNet | Official implementation | 256 × 256 | 130 | 12 | Adam | $1 \times 10^{-4}$ | BCE |
| HiFormer | amirhossein-kz/HiFormer | Author-released implementation | 256 × 256 | 130 | 12 | Adam | $1 \times 10^{-4}$ | BCE |
| UNeXt | jeya-maria-jose/UNeXt-pytorch | Official implementation | 256 × 256 | 130 | 12 | Adam | $1 \times 10^{-4}$ | BCE |
| SSW-OCTA | ShellRedia/Snake-SWin-OCTA | Official implementation | 256 × 256 | 130 | 12 | Adam | $1 \times 10^{-4}$ | BCE |
| RCNet | Implementation developed in this study | Proposed method | 256 × 256 | 130 | 12 | Adam | $1 \times 10^{-4}$ | BCE |

**Notes:** All models were independently reproduced and trained using the same training, validation, and test partitions. The original OCTA images were resized to 256 × 256 pixels and represented in three-channel format. Horizontal/vertical flipping and rotation were applied only to the training set. The model checkpoint with the highest validation DSC was selected for test evaluation. A probability threshold of

0.5 was applied to obtain the final binary segmentation mask. Architecture-specific operations were retained according to their respective public implementations, while the input pipeline and output heads were adapted for binary OCTA capillary segmentation.
