# Supplementary material for: RCNet: A Hybrid Network Adapted for OCTA Retinal Capillary Segmentation
Source: Bioengineering (Basel). 2026 Sep 30;13(10):1147. doi: 10.3390/bioengineering13101147 (PMC13701182; doi:10.3390/bioengineering13101147)
Supplement: Supplementary file 1 [file bioengineering-13-01147-s001.zip › Supplementary Table S2.pdf]

Bootstrap analysis of OCTA-500 test results.

To assess the uncertainty of the reported OCTA-500 results, we analyzed DSC and clDice scores for RCNet and the two closest-performing comparators, OCTA-Net and SSW-OCTA, on the same 60 test images. Each metric was calculated for individual images and then averaged across the test set. We generated bootstrap samples by resampling independent test cases with replacement. The same cases were selected for all three models in each resample. For each model, we calculated a 95% percentile confidence interval for its mean score. We also calculated paired confidence intervals for the differences between RCNet and each comparator by subtracting their mean scores within each resample.

Table S2. Image-level performance and paired bootstrap comparisons on OCTA-500 test set.

**Model**	**DSC, % (95% CI)**	**clDice, % (95% CI)**	**RCNet − method: DSC, percentage points (95% CI)**	**RCNet − method: clDice, percentage points (95% CI)**
RCNet	60.47 (58.79, 62.21)	59.39 (57.66, 61.16)		
OCTA-Net	60.03 (58.24, 61.88)	57.61 (55.74, 59.54)	+0.44 (−0.49, +1.35)	+1.78 (+0.98, +2.56)
SSW-OCTA	59.84 (57.99, 61.80)	57.15 (55.41, 58.94)	+0.63 (−0.22, +1.43)	+2.24 (+1.35, +3.15)

Note: DSC and clDice are reported as percentages. Paired differences are calculated as RCNet minus the comparator and reported in percentage points. Confidence intervals are obtained by resampling the same test cases for all models. This analysis describes uncertainty associated with the sampled test cases for the evaluated trained models; it does not quantify variation across independent training runs.
